# Assessing developability early in the discovery process for novel biologics

**DOI:** 10.1080/19420862.2023.2171248

**Published:** 2023-02-23

**Authors:** Monica L. Fernández-Quintero, Anne Ljungars, Franz Waibl, Victor Greiff, Jan Terje Andersen, Torleif T. Gjølberg, Timothy P. Jenkins, Bjørn Gunnar Voldborg, Lise Marie Grav, Sandeep Kumar, Guy Georges, Hubert Kettenberger, Klaus R. Liedl, Peter M. Tessier, John McCafferty, Andreas H. Laustsen

**Affiliations:** a Center for Molecular Biosciences Innsbruck (CMBI), Department of General, Inorganic and Theoretical Chemistry, University of Innsbruck, Innsbruck, Austria; b Department of Biotechnology and Biomedicine, Technical University of Denmark, Kongens Lyngby, Denmark; c Department of Immunology, University of Oslo, Oslo, Norway; d Department of Immunology, University of Oslo, Oslo University Hospital Rikshospitalet, Oslo, Norway; e Institute of Clinical Medicine and Department of Pharmacology, University of Oslo, Oslo, Norway; f Authera AS, Oslo Science Park, Oslo, Norway; g National Biologics Facility, Department of Biotechnology and Biomedicine, Technical University of Denmark, Kongens Lyngby, Denmark; h Biotherapeutics Discovery, Boehringer Ingelheim Pharmaceuticals Inc, Ridgefield, CT, USA; i Roche Pharma Research and Early Development, Large Molecule Research, Roche Innovation Center Munich, Penzberg, Germany; j Department of Chemical Engineering, Pharmaceutical Sciences and Biomedical Engineering, Biointerfaces Institute, University of Michigan, Ann Arbor, Michigan, USA; k Department of Medicine, Cambridge Institute of Therapeutic Immunology and Infectious Disease, University of Cambridge, Cambridge, UK; l Maxion Therapeutics, Babraham Research Campus, Cambridge, UK

**Keywords:** Biologics, developability, antibodies, drug development, biotherapeutics, drug discovery, drug properties, half-life, immunogenicity, manufacturability

## Abstract

Beyond potency, a good developability profile is a key attribute of a biological drug. Selecting and screening for such attributes early in the drug development process can save resources and avoid costly late-stage failures. Here, we review some of the most important developability properties that can be assessed early on for biologics. These include the influence of the source of the biologic, its biophysical and pharmacokinetic properties, and how well it can be expressed recombinantly. We furthermore present *in silico, in vitro*, and *in vivo* methods and techniques that can be exploited at different stages of the discovery process to identify molecules with liabilities and thereby facilitate the selection of the most optimal drug leads. Finally, we reflect on the most relevant developability parameters for injectable versus orally delivered biologics and provide an outlook toward what general trends are expected to rise in the development of biologics.

## Introduction

**Figure 1.**
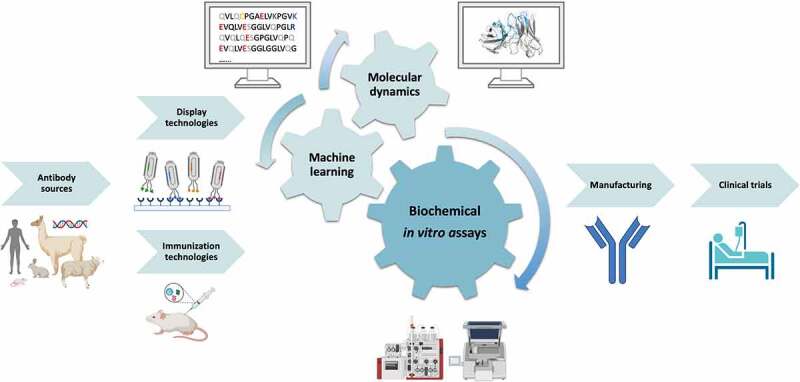
**Schematic overview of antibody sources, discovery strategies, *in vitro* assays, and *in silico* methods used to assess developability properties during the development of biologics**. Antibodies can be of either natural or synthetic origin and are typically discovered using various display technologies or immunization strategies, or a combination of these. In addition, antibodies can be derived from patient populations. During the development of antibodies, various *in vitro* assays in combination with *in silico* methods for machine learning and molecular dynamics can be applied to assess the antibodies’ developability properties and select candidates with the best developability profile.

Bringing a biologic from early discovery to a marketed product is an immensely expensive endeavor, with the average investment being further compounded by the high attrition rates in clinical development.^1^ While the clinical success of a biologic ultimately depends on its safety and efficacy in human patients, the underlying properties of these endpoints include everything from drug potency, specificity, immunogenicity, and pharmacokinetics to biophysical behavior *in vivo* and during storage.^2,3^ Moreover, with more advanced biologics such as bispecific antibodies and antibody-drug conjugates in the pipeline, chemistry, manufacturing, and control (CMC) and regulatory aspects can be key to commercial success, when the biologic must be repeatedly produced at consistently high quality.^4^ Combined, these performance measures are often referred to as a biologics’ “developability”.^5^ In this review, the different developability parameters for biologics will be presented together with a discussion on how to assess and optimize these using both *in silico, in vitro*, and *in vivo* methods (Figure 1). When optimally employed, the assessment and improvement of developability properties can lead to lower attrition rates, as well as improved manufacturability, enabling the production of higher quality, lower cost biologics for the benefit of patients worldwide.

## Antibody sources

Antibodies and antibody fragments comprise the largest group of biologics.^6^ Antibodies are typically sourced from blood plasma,^7^ hybridomas,^8^ or recombinant cell lines,^9^ and originate from natural (animal or human) or synthetic sequences. Both the source and the origin may affect the antibodies’ developability profile. Natural antibodies, which are derived from natural, non-engineered sequences and typically sourced from (immunized) animals or patient populations, are to date the most commonly used source of antibodies.^7^ Such antibodies inherently carry the benefit of having been derived from a living creature and have gone through affinity maturation and self-tolerance *in vivo*, which often results in a good developability profile.^7^ However, in the context of clinical use, there is a stark difference between human and animal-derived antibodies. Whilst the former constitutes antibodies that have a low risk of immunogenicity, antibodies of non-human origin may elicit severe immune responses in patients, depending on the animal from which they were derived. The risk of immunogenicity can be reduced in several ways. For example, antibodies can be humanized or animals transgenic for human immunoglobulins (e.g., rabbits^10^ or mice^11–14^) can be used for immunization. A potential limitation of antibodies derived from immunization campaigns is that they will primarily target the most immunogenic epitopes of an antigen. This can limit epitope diversity and potentially prevent the discovery of binders to important, but low- or non-immunogenic antigen epitopes. Furthermore, inadequate immunization strategies can prevent antibodies from being discovered toward targets that are highly homologous to targets expressed within the immunized host due to self-tolerance.

To avoid these limitations, other types of antibodies have increased in popularity, *i.e*., synthetic antibodies,^15,16^ as they allow the discovery of binders toward, in principle, any target. Such antibodies may originate from rationally designed recombinant antibody repertoires and are typically discovered via *in vitro* display technologies.^17^ Using such methodologies grants substantial control over the antibody libraries. For example, certain amino acid residues known to be detrimental to binding interactions can be removed, lengths of variable regions can be adjusted, new formats can be experimented with, and overall antibody libraries with variability beyond nature can be created.^18^ As a result, this allows for their use against a substantially larger target space. Synthetic antibodies, however, typically come with the drawback of not having undergone *in vivo* maturation and being derived from a natural origin. Thus, they may potentially carry unexpected developability issues, such as unwanted polyreactivity, immunogenicity, propensity for aggregation, and poor expression profiles. Nevertheless, in combination with sophisticated display technologies and advanced computational tools, these risks can be minimized.

## Advances in display technologies

Over the past few decades, display technologies have permitted the creation of large and diverse populations of antibodies and other proteins and peptides, from which individual variants with desired target-binding properties can be isolated.^19^ The requirements for generating an antibody drug, however, do not solely revolve around the binding specificity or affinity of the antibody. Other factors can affect the likelihood that a lead candidate antibody can be developed into an efficacious, manufacturable, safe, and stable drug. For example, the propensity of the antibody to aggregate upon formulation at higher concentration, the extent to which non-specific interactions occur, and the pharmacokinetics of the antibody can lead to the failure of a lead molecule during the development process.^3,5,20^

In applying display technologies, populations of antibodies may be derived from re-arranged antibody genes from B cells of human and non-human donors, constructed from synthetic antibody genes where variation has been introduced during oligonucleotide synthesis or a combination of both.^21^ Developability problems can arise irrespective of the starting source and even “natural”, immune-derived antibodies may suffer from biophysical liabilities. It has also been shown that engineering of lead antibodies with a sole focus on improving affinity can generate affinity-enhanced variants with poorer biophysical properties.^22,23^ For example, Buchanan et al.^24^ identified an unpaired cysteine, and Dobson et al.^22^ used hydrogen deuterium exchange to identify hydrophobic residues (W30 and F31 in VH complementary-determining region (CDR)1 and L56 in VH CDR2), which were protected in the initial dimerization preceding aggregation. Alternatively, structures (or structural models) could be used to identify hydrophobic or positively charged patches as used by Bethea et al.^25^ (F99, H100 and W100a in VH CDR3) and Dyson et al.^23^ (S52, F54, and R57 in VH CDR2). Based on these approaches, individual variants can be produced and assessed for improved biophysical characteristics. In some cases, the developability issues of clones can be solved by identifying problematic regions (*e.g.*, hydrophobic patches), producing individual variants, and assessing these for improved biophysical characteristics.^22,24,25^

Historically, the consideration of developability issues has been addressed after the initial discovery, affinity optimization, and selection of an antibody drug lead for preclinical development. Increasingly, drug developers are assessing biophysical characteristics earlier in the discovery process, recognizing that this will avoid losses in time and money later. Rather than waiting to screen tens to hundreds of output clones for biophysical characteristics, the ability to create large display libraries of variants and select directly for good biophysical characteristics would greatly facilitate the search for antibody variants with optimal properties.

The power of selection technology, best exemplified by display on bacteriophage (phage display),^26^ has been applied to selecting clones for greater thermostability^27^ by exposing libraries of clones to elevated temperatures and selecting for retained binding to protein A or protein L, which requires correct folding.^28^ Others have subjected libraries of antibodies to acidic conditions to identify again clones that combined thermodynamic stability and aggregation-resistant unfolded states.^28^

While selection of clones with low thermostability will help identify aggregation propensity, there may be clones with normal melting temperature where biophysical problems only emerge when the antibody is concentrated (*e.g*., to 10–100 mg/mL as required for subcutaneous administration). Therefore, additional selection methods are needed. The principle underlying the power of phage display (coupling an encoding gene to its displayed product) has also been applied to other display systems using baculovirus,^29^ ribosomes,^30^ yeast,^31^ and higher eukaryotes, such as mammalian cells.^29,31^ Mammalian display, in particular, appears to offer benefits over the other systems. Using a mammalian display system^23^ showed that closely related clones, with differing biophysical properties, can be distinguished based simply on display levels. The underlying mammalian display system, first described by Parthiban et al.,^32^ achieves transcriptional normalization by integrating antibody genes into a single genome locus. Thus, display levels are determined by the properties of the antibody itself, rather than variable transcriptional activity. In contrast to secretion-based systems, the expressed antibodies are retained on the cell surface via a transmembrane domain and achieve high local concentrations in the endoplasmic reticulum or cell surface. Antibodies are therefore exposed to high concentrations and clones with poor biophysical properties are likely to aggregate at such high surface concentrations, with aggregates presumably removed by the quality control machinery of the cell. In turn, this results in low presentation levels of such aggregation-prone or “sticky” clones.

This ability to detect biophysical properties by mammalian display then permitted selection of clones with improved developability profiles from libraries of variants.^23^ In practice, libraries were created, and the clones with highest levels of presentation were selected. Multiparametric flow cytometry allows simultaneous screening for optimal biophysical properties while retaining antigen binding, and thereby allows paratopic residues involved in target binding to be addressed for improved biophysical properties while retaining target binding. The power of such a system was exemplified by creating variants of an anti-PCSK9 antibody with superior biophysical properties and reduced immunogenicity compared to the parental antibody (bococizumab). These and other experiments thereby demonstrate how *in vitro* display technologies can be fine-tuned for the discovery of antibodies with improved developability profiles *ab initio*.

## Biophysical characterization

Irrespective of which strategy is used for antibody discovery, there typically are several early-stage candidates, ranging from tens to thousands, that must be reduced to only one candidate for cell line development and manufacturing. To select the optimal candidate, an increasing amount of attention is being paid to biophysical characterization of therapeutic antibodies in addition to their functional properties, which enables the deselection of antibodies with poor developability properties early in the drug development process. There are multiple developability assays to facilitate this selection, which have been reported to correlate to different extents with attrition rates of clinical-stage antibodies.^2,3^

One key feature of antibody drugs is their high specificity, defined by high on-target binding and low off-target and non-specific binding, which is important to reduce the risks of abnormal pharmacokinetics and fast antibody clearance.^33,34^ To evaluate non-specific binding, enzyme-linked immunosorbent assays (ELISAs), which typically involve binding to multiple non-targets, such as single-stranded DNA, double-stranded DNA, lipopolysaccharide (LPS), insulin, and keyhole limpet hemocyanin (KLH), have been most commonly used.^3,35–37^ For increased sensitivity, assays such as the polyspecificity particle (PSP) assay^38^ can be used, which involves detecting the binding of either complex antigen mixtures (*e.g*., soluble membrane proteins from Chinese hamster ovary (CHO) cells) or defined protein reagents (*e.g*., ovalbumin) to immobilized antibodies on Protein A coated beads via flow cytometry. Similar to the PSP assay, other assays also use complex antigen mixtures, including baculovirus particles,^39^ whole cells,^38^ and cell lysates (polyspecificity reagent, PSR).^40^ PSR has been shown to correlate quite well with cross-interaction chromatography (CIC).^41^ Thus, another way to study non-specific interactions is chromatography. In CIC, non-specific protein interactions, such as monoclonal antibodies interacting with immobilized polyclonal antibodies, are detected via their relative retention times.^42–44^ A related chromatography method, namely standup monolayer chromatography (SMAC), instead detects non-specific interactions between monoclonal antibodies and the column.^45^ For example, heparin chromatography has been described for identifying antibodies with abnormal pharmacokinetics via increased cell-surface interactions, leading to excessive pinocytosis.^46^ In addition to specificity, the SMAC measurements identify antibodies with increased likelihood of precipitation and aggregation. Non-specific binding can also be induced by surface hydrophobicity, which is often quantified using hydrophobic interaction chromatography (HIC).^47,48^

Another key feature of antibody drugs is their high colloidal stability and low propensity to self-association and aggregation, which is especially important for concentrated liquid formulations used for subcutaneous delivery.^49^ Several assays have been reported for evaluating antibody self-association, including static and dynamic light scattering.^50,51^ While these assays have proven to be valuable, they are not compatible with early-stage development due to their requirement for high antibody concentrations and purity. Therefore, alternative assays have been developed, including self-interaction chromatography (SIC),^52,53^ CIC,^41,43^ and clone self-interaction by biolayer interferometry,^44^ the latter of which has been shown to correlate with SIC and CIC. To enable even higher throughput and the use of low antibody concentrations, two nanoparticle-based assays have been reported, affinity-capture self-interaction nanoparticle spectroscopy (AC-SINS)^54–57^ and charge-stabilized self-interaction nanoparticle spectroscopy (CS-SINS).^50^ AC-SINS is most commonly performed in a solution mimicking physiological conditions (pH 7.4, phosphate-buffered saline),^3^ and its measurements have most commonly been linked to pharmacokinetic properties,^33,58^ although it has also been used for formulation applications.^59^ CS-SINS is performed in a common formulation condition (pH 6, 10 mM histidine) and has been reported to identify antibodies with low viscosity and opalescence in concentrated antibody formulations.^50^

A third key feature of therapeutic antibodies is their high folding stability. Given the goal that formulated antibodies have a shelf-life of several years, obtaining real-time stability data are extremely time-consuming. To accelerate stability analysis, various stress conditions are commonly used. Thermal stability is typically measured using differential scanning calorimetry or differential scanning fluorimetry.^60^ In addition to temperature, surface-mediated stress can be used to evaluate antibody stability. For example, the recently described hydrophobic nanoparticles surface-stress assay was used with 14 antibody variants spanning a range of solubility values to identify variants characterized by high instability against agitation in the presence of air–water interfaces. Furthermore, aggregation assessment by this surface-mediated stress assay correlated well with other approaches to assess biophysical properties, such as temperature-induced aggregation and AC-SINS.^61^ Taken together, these and other *in vitro* assays allow the drug developer to select antibodies with combinations of preferable developability features. However, all *in vitro* assays require the expression of antibodies, sometimes including purification and formulation, followed by experiments to be carried out in the laboratory. To enable screening of an even higher number of antibodies, reduce cost, manual labor, and the requirement for antibody proteins, *in silic*o methodologies are now gaining increased attention and multiple computational strategies to address developability exist, and additional ones are being rapidly developed.

## Big data, machine learning, and computational assessments of developability

**Figure 2.**
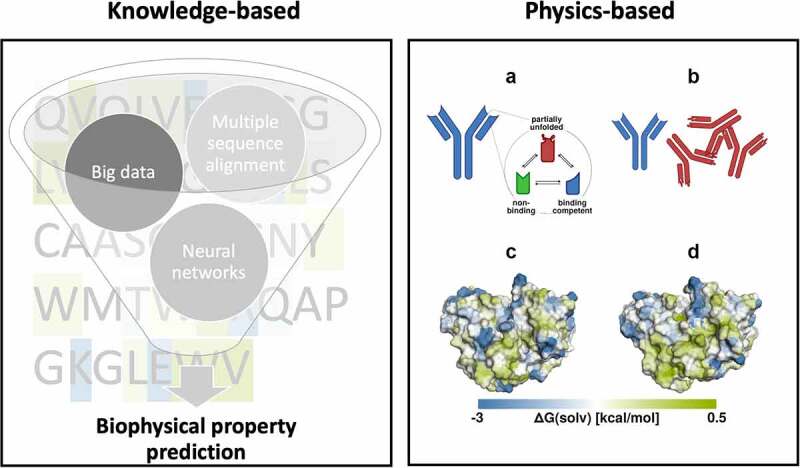
**Knowledge and physics-based approaches for characterizing biophysical properties of antibodies**. Left panel, knowledge-based: Overview of critical steps for sequence-based *in silico* prediction of biophysical properties from several thousands of potential hit sequences. Right panel, physics-based: A) The antibody binding interface exists as an ensemble of conformations, which includes binding competent as well as non-binding states. Partially unfolded conformations also exist with a lower probability. B) Different conformations exhibit different properties, where partially unfolded conformations may aggregate which leads to further unfolding. In C) and D), the hydrophobicity profile of two different conformations of the TNF-α binding antibody golimumab is mapped on its molecular surface using localized free energy of hydration. The two conformations show a significantly altered hydrophobicity profile and will therefore most likely interact differently with other hydrophobic molecules.

Computational assessments, which play an important role in assessing developability of biologics, are particularly useful in the early stages of biotherapeutic drug discovery where usually little to no experimental data is available.^62–66^ For example, an ideal stage for applying the computational assessments is immediately after sequencing the fragment variable regions (Fvs) of the antibody binders obtained from immunizations or display experiments, where there is a need to select a subset of binders for further experiments (Figure 2). Typical strategies for this selection include clustering of antibodies with respect to diversity of VH-VL germline pairs and epitope/paratope diversity. Inclusion of developability assessments at this stage is important to ensure focused use of available experimental resources, given that immunization campaigns often yield several thousands of potential hit sequences. Large portions of the encoded antibodies may either not bind in the subsequent confirmatory experiments or show multiple developability challenges. Flagging such antibodies via computational analyses helps prioritize antibodies for experimental testing (Figure 2). The developability assessments at this stage can include both sequence and structure-based methods or a combination thereof.^63,64,67,68^

Typical components of developability assessments should include detection of motifs for chemical degradation, such as oxidation, deamidation, Asp isomerization, and along with those, motifs for glycosylation and presence of non-canonical Cys residues. Percent humanness (or “naturalness”),^69^ potential aggregation-prone regions and MHC class II binding immune epitopes can also be predicted using sequence-based methods.^64,70,71^ Advances in structural prediction of antibody variable regions in recent years have enabled fast and reliable high-throughput modeling of the antibody variable regions on a regular basis.^72–76^ Nevertheless, recent contributions on understanding the conformational behavior of CDR-H3 and its prediction indicate that the need for better models still exist.^72,77,78^ It is now feasible to model hundreds or even thousands of antibody structures with moderate computational resources. These Fv models can be used to compute several physicochemical descriptors, such as isoelectric point (pI), charge, hydrophobic imbalance, surface areas buried at the VH-VL interface along with molecular surface patches.^64,79,80^ These descriptors describe electrostatic as well as hydrophobic properties of the antibodies along with their conformational stabilities.

The pI of an antibody is an important property that potentially impacts the developability in multiple ways. It can affect various aspects, including those related to antibody purification, formulation (*e.g*., stability and viscosity), and pharmacokinetic properties. Generally, the isoelectric point of therapeutic antibodies is between 6 and 9. However, various developability challenges have been reported for some antibodies with relatively low (pI <6.5-7) or high (pI >8.5-9) isoelectric points.^2,36,81–84^ For example, Bailly et al. revealed that increasing the initially low pIs (pIs 6.3-6.7) of humanized antibodies to higher values (pIs >7) improved their purification yields and reduced their tendency to aggregate.^2^ Furthermore, the antibody pI affects pharmacokinetics, tissue distribution,^81^ and binding to FcRn and thereby the antibody half-life.^82^ By comparing the computed properties of the newly discovered confirmed binders (hits) with those of the antibodies in clinic or those currently available in the market,^3,63,79,80^ one can profile drug-likeness of the hits.

Among the antibodies chosen for experimental testing, the computational developability assessments can help identify potential lead molecules that possess an optimal combination of functional as well as physicochemical attributes. However, depending on peculiarities of individual drug discovery projects, these lead molecules may need to be affinity matured,^85,86^ humanized,^87–89^ formatted in novel molecular constructs,^90^ and further optimized for fitness with development platforms. This calls for the use of computational protein engineering tools to optimize the lead molecules. If the final molecular format of the lead molecule is a monoclonal IgG antibody, then computational engineering of the variable regions for improved developability attributes is more likely to translate into experimentally verifiable results and, surprisingly, only a few mutations can make substantial improvements in the CMC properties of the drug candidates.^68,91–93^

Once one or a few optimal lead molecule(s) have been selected, the discovery project then moves into the early development phase. In this phase, the optimized lead candidates are assessed for their fitness with the development platform(s). Computational developability assessments should now involve modeling the full-length antibody structure for a more accurate description of the computed properties and their agreement with the standard development experiments that involve larger amounts of materials and larger sample volumes. Use of multi-scale molecular simulations and machine learning models at these stages can help with identifying early formulation process challenges, such as aggregation, diffusion interaction, viscosity and solubility,^94–104^ physicochemical degradation,^105–107^ and immunogenicity.^108,109^ Specifically, use of explicit solvent molecular dynamics (MD) simulations can potentially provide a molecular-level understanding of molecular response to thermal and other stresses.^110^ Expanding the scope of such simulations to include the considerations of formulation buffers, salt, pH, and excipients will pave the way toward *in silico* formulation development for biologics. Finally, while most sequence and developability reports are based on biophysical models, machine learning methods for predicting developability parameters are being developed that infer developability parameters from the antibody sequence without the need for structural modeling.^111,112^

While the above-discussed machine learning approaches are discrimination tasks (*e.g*., prediction of variable X), deep learning also allows for the possibility of “generative machine learning”, which consists in learning the underlying features of a training dataset of antibody sequences with a specific property and then generating new antibody sequences, different from the training dataset, but with similar properties (also called features) as in the training dataset, where features may consist of developability parameters,^113^ binding parameters, or both.^69,114–116^ Interestingly, using a simulation framework, it was recently shown that generative learning can explore new binding and developability spaces.^114^ However, it remains to be determined to what extent out-of-distribution (generation of antibody sequences with features that were not included in the training dataset) learning is feasible and how much training data is necessary for achieving this task.^64^ In addition, so far, no antibody-focused generative approaches exist that would allow the generation of antibodies with multiple pre-specified features.^117,118^ Large-scale simulations may help in understanding the minimal data complexity necessary for such tasks.^114,119–122^ In summary, computation plays an important role in assessing the developability of biologics.

## Biophysical properties of biologics from structure and molecular dynamics

Structural and dynamic characterization of antibodies is a prerequisite for engineering properties, such as chemical modifications, antigen recognition and receptor binding.^123,124^ The three-dimensional structure of proteins, in particular antibodies, is not static, but fluctuates constantly.^125,126^ These fluctuations can occur on different timescales, ranging from the low nanosecond timescale up to seconds.^127^ Even rare conformations can be relevant if they lead to a modification that is irreversible or part of a one-sided equilibrium, for example in aggregation or chemical modifications (Figure 2A).^128–130^ Furthermore, several studies have used molecular dynamics simulations to estimate the thermal stabilities of antibodies. For instance, the fraction of native contacts computed from simulations at high temperature has been shown to correlate with experimental melting temperatures.^131^

To elucidate the function and properties of antibodies, single-static structures are not always sufficient and thus, the antibody paratope may rather be characterized as conformational ensembles in solution.^125,127,132^ These paratope ensembles have been described by correlated CDR loop movements and interdomain and elbow angle rearrangements.^125,133,134^ This high flexibility and conformational diversity of the antigen-binding site, in particular of the CDR-H3 loop, challenges antibody structure prediction.^132,135^ Despite the substantial advances in antibody structure prediction,^72–76^ it is critical to carefully evaluate the structure model before further processing, as some of the models can contain structural inaccuracies, substantially deteriorating biophysical surface property predictions.^136–138^ Special care has to be taken when predicting antibody structures based on apo X-ray structures, which can be distorted by crystal packing effects and consequently do not correspond to the dominant solution-structure.^125,132,139^

Accounting for the high conformational diversity of antibodies by considering them as ensembles in solution can facilitate not only structure prediction but also guide the engineering workflow, facilitate identification of developability liabilities and optimize biophysical properties.^136,140^ For instance, it has been shown that aggregation of antibodies is accelerated by low-population states (Figure 2A-B) that become more frequent near hydrophobic surfaces and at phase boundaries.^141^ The hydrophobic interaction with the surface leads to a conformational shift toward more hydrophobic conformations, which in turn are more prone to aggregate (Figure 2A-B). In the same way, elevated temperatures shift the ensemble toward aggregation-prone conformations. This effect is experimentally seen as an irreversible aggregation.^128,142,143^ Therefore, it is necessary to describe antibody properties as an ensemble of structures. Molecular dynamics simulations^127,144^ provide such an ensemble in solution, thereby increasing the probability that the conformations responsible for hydrophobic or aggregation behavior are included (Figure 2C-D). Several works have studied the effect of conformational ensembles on hydrophobicity. While more coarse methods based on hydrophobicity scales are in general less sensitive to structural differences,^145,146^ a study based on explicit solvent thermodynamics found a critical influence of input structures and side chain orientations.^136^

It has been shown that both binding and biophysical properties^46^ of antibodies can be charge-dependent. However, the pKa of amino acids in proteins remains highly challenging to predict. In contrast to empirical methods such as protein pKa calculation,^147^ which assign protonation states for a single structure, constant pH molecular dynamics allows for the incorporation of protonation changes in structural sampling.^148,149^ Capturing the changes in protonation is particularly important for designing pH responsive antibodies, for example targeting acidified tumor microenvironments.

Thus, we strongly suggest considering antibodies as conformational ensembles in solution, which can improve structure prediction and allow assessment of biophysical properties that facilitate the development of antibody therapeutics.

## Cellular assays for developability assessment

**Figure 3.**
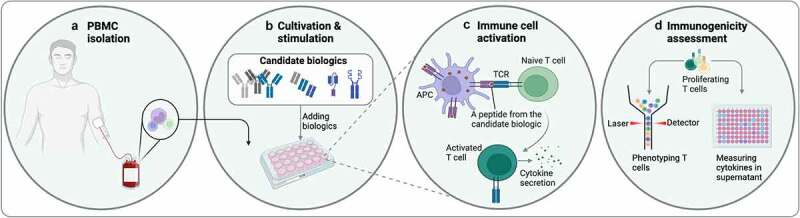
**Schematic illustration of a Peripheral Blood Mononuclear Cell (PBMC) immunogenicity assay**. A) PBMCs are isolated from healthy donors. B) Isolated cells are cultured in cell media with added candidate biologics. C) Candidate biologics are taken up by antigen presenting cells (APCs) and presented to T cells in culture. If the biologic is immunogenic, this may lead to T cell activation and concurrent cytokine secretion. D) Immunogenicity assessment can then be performed by phenotyping the T cells following stimulation with candidate biologics and measuring cytokine levels in culture supernatant.

To a patient’s immune system, therapeutic biologics are foreign molecules, and therefore they may be immunogenic. Immunogenicity arising from structural traits and formulations can trigger both acute and long-term issues, such as innate and adaptive immune activation,^150^ acute cytokine storms,^151^ or a rise of anti-drug antibodies (ADA) causing neutralization of the drug and loss of therapeutic efficacy.^152,153^ Complex formation between ADA and the biologic can have detrimental effects to patients,^154^ which may be tied to an innate immune response leading to antigen presentation, secretion of inflammatory cytokines, and activation of T and B cells.^155^ Depending on the type of immune cells involved, immune activation may or may not lead to ADAs.^155–157^ A driver for antibody immunogenicity is T cell activation and subsequent cytokine release.^158,159^ This may be addressed *in vitro* by the use of immune cell activation assays, where pooled peripheral blood mononuclear cells are exposed to candidate biologics to reveal the presence of activating T cell epitopes.^158^ The general principle of such assays is illustrated in Figure 3. This type of assay has been shown to correlate with clinical immunogenicity of monoclonal IgG antibodies by revealing increased T cell proliferation and release of pro-inflammatory cytokines such as IL-2 and IFN-γ,^160^ and have been highlighted as important tools by both the European Medicines Agency and the US Food and Drug Administration.^161,162^ While such tools are often used to de-risk pre-clinical development, the in-patient immunogenicity of biologics remains difficult to predict, likely due to variations in drug delivery, the therapeutic context in which a given biologic is used, as well as the complexity of the human immune repertoire, including individual haplotype of human leukocyte antigens.

Both IgG antibodies and albumin-based biologics have favorable transport properties within and across barriers, providing them with a long plasma half-life of three weeks on average in humans. This arises from their ability to bind to the neonatal Fc receptor (FcRn), which resides predominantly in acidified endosomes in a multitude of both non-hematopoietic and hematopoietic cell types.^163,164^ Here, FcRn encounters its ligands following their cellular entry by fluid-phase pinocytosis and binds to them in a pH-dependent fashion, where binding occurs at mildly acidic pH, and no binding or release occurs at neutral pH. This directs cellular recycling or transcytosis, and as such, FcRn engagement rescues the ligands from intracellular degradation, which results in high blood concentrations and long plasma half-life. The efficiency by which different biologics undergo this process has an enormous impact on their pharmacokinetic properties and biodistribution. This has spurred establishment of biophysical methods to determine pH-dependent binding kinetics toward FcRn by, for instance, the use of surface plasmon resonance, microscale thermophoresis, and affinity chromatography techniques, with the aim to predict the efficiency of FcRn-mediated transport.^165–168^ While these accessible and high-throughput biophysical methods can provide information regarding binding at a given pH condition, they typically do not unravel how the molecules bind FcRn throughout a pH gradient, and therefore do not directly mimic a cellular setting. However, the pH gradient can be mimicked by analytical FcRn affinity chromatography, where a gradual increase in pH is used to address dissociation of FcRn-targeted molecules from the receptor coupled to the matrix.^166,169^ While such studies can reveal valuable information, caution should be taken, as they do not account for the fact that FcRn is embedded in a negatively charged cell membrane and follows transport pathways involving different endosomal structures.^170–172^ Furthermore, they do not account for the diversity by which different cell types use FcRn for both ligand transport and antigen presentation and processing of immune complexes in concert with Fcγ receptors.^164,173–178^ In addition, the stoichiometry of FcRn in complex with both IgG and albumin in a cellular context is complex and far from fully understood.^179–181^ Thus, a need remains for reliable assays that can be used to dissect the determinants of cellular handling of IgG and albumin-based formats in both a FcRn-independent and dependent manner. This type of insights may guide lead selection by identifying so-called “red flags”, including, for example, polyreactivity, stability, and unfavorable pharmacokinetics early in the discovery process.^3,79,80,182^ These traits arise from biophysical properties such as surface charge, charge patches, isoelectric point, and hydrophobicity, and affect both FcRn binding, cellular transport, and *in vivo* performance.^80,168,169,183^ Therefore, cellular assays mimicking these traits are vital, and may provide necessary data input for computational analysis.^3,80^

**Figure 4.**
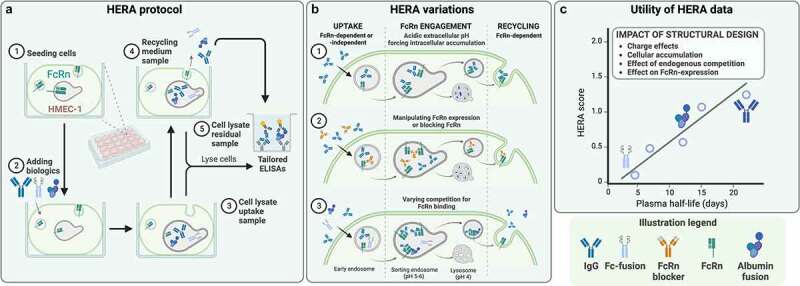
**Human Endothelial Recycling Assay (HERA) as a tool for *in vitro* pharmacokinetic assessment and addressing FcRn-targeting strategies**. A) Generalized HERA protocol. (1) Stably FcRn-transfected human microvascular endothelial cells (HMEC)-1 are seeded, prior to (2) adding FcRn-binding candidate biologics to two parallel cell plates. Following an incubation period, (3) cells from one plate are lysed to obtain an uptake sample. For the other plate, the media is exchanged to recycling medium, and after another incubation period, (4) the medium is harvested as a recycling sample, and (5) the cells are lysed to obtain a residual sample. Candidate biologics in all samples are quantified by an ELISA tailored for specific detection of the assessed biologic. B) Variations of the HERA protocol, enabling analysis of both FcRn-dependent and -independent uptake, cellular accumulation, and FcRn-dependent recycling. Variations include (1) performing the uptake step at mildly acidic extracellular pH, effectively forcing intracellular accumulation of biologics by preventing FcRn-mediated recycling, (2) manipulating FcRn-expression or blocking binding to FcRn to analyze FcRn-dependent and -independent cellular accumulation, and (3) introducing competition for FcRn binding to mirror endogenous competition on the ligand-binding sites of FcRn and its effects on the cellular transport for FcRn-binding biologics. C) HERA data can be used to address the impact of structural design of candidate biologics on FcRn-mediated cellular transport and unspecific cellular accumulation. For some candidate biologics, HERA data may allow for calculation of a score that correlates with plasma half-life in human FcRn-transgenic mice.

Indeed, cellular assays addressing both FcRn-mediated cellular recycling and transcytosis exist and have been used to reveal insights on how FcRn-engaging formats are taken up and sorted.^84,165,173,174,183–188^ These assays have further been used to predict *in vivo* characteristics, exemplified by correlations between cellular transport properties and plasma half-life or clearance.^84,165^ The assays can include advanced live cell imaging to track both the receptor and its ligands and yield valuable mechanistic insights.^171,189,190^ However, while useful and yielding high resolution, imaging relies on protein labeling, which may affect overall stability as well as FcRn binding and transport.^184,191^ While many reports focus on measuring the amounts of molecules transported out of, or across cells, the rate of intracellular accumulation and degradation should also be accounted for. The importance of considering these parameters is exemplified by the use of a human endothelial cell-based recycling assay (HERA), where the cells overexpress human FcRn.^165^ HERA can be used for screening of the ability of FcRn-targeted molecules to be taken up, followed by their rescue from intracellular accumulation and/or degradation (Figure 4A). Notably, HERA only requires small amounts of proteins, and there is no need for labeling, as the parameters can be measured by ELISA setups on collected samples. Furthermore, the assay allows for manipulation of pH and blocking of the ligand binding sites, as well as modulation of the receptor expression level,^165,168,183^ which enables tailoring of the assay to specific needs and questions (Figure 4B). This provides a broad utility, and the cellular readout correlates with *in vivo* data. For example, HERA screening of IgG1 Fc-engineered variants with distinct FcRn binding kinetics revealed a predictive correlation with their plasma half-life in human FcRn transgenic mice (Figure 4C).^165^ In a recent study, HERA was used to address the efficiency by which different IgG1 Fc-containing biologics undergo cellular recycling, which, in combination with studies in transgenic mouse models, hints at the underlying reasons for the observably short half-life of IgG1 Fc-fusions compared to monoclonal IgG1 antibodies with corresponding specifities.^168^ Importantly, the study also addresses the discrepancy between recycling and transcytosis, which may be revealed by combining recycling assays with transwell studies.^84,168,186^ The latter allows for quantification of FcRn-mediated transcytotic transport, which, as opposed to recycling, correlates with increased clearance.^84^

To realize the full utility of the cellular assays described above, the biophysical properties of biologics need to be considered. Factors such as the surface charge of Fc-fused structures may affect the cellular handling of biologics in both FcRn-dependent and independent manners. For example, the antibodies briakinumab and ustekinumab bind to the same target, the p40 subunit of IL12/23, but their varying charge profiles confer vastly different half-lives in humans.^169,183,192,193^ Specifically, the charge of the briakinumab Fv alters its interaction with FcRn.^169,183,194^ Cellular studies have revealed that this causes FcRn-independent cellular accumulation of briakinumab, which explains its shorter half-life.^183^ Interestingly, the same study found that the half-life of briakinumab can be improved by Fc-engineering, exemplifying that modulating the core binding to FcRn may adjust for unfavorable biophysical traits of Fc-fused structures. It also exemplifies the complexity tailored cellular assays may reveal. These are just a few of many existing examples of how distal structural traits may affect Fc binding to FcRn.^164,194,195^ Furthermore, HERA and other cellular assays can also be used to address transport of albumin-based molecules, which reflect their *in vivo* pharmacokinetic properties and ability to cross polarized mucosal epithelial cell surfaces.^165,167,184,196^

## Preclinical pharmacokinetic assessment in mice

While cellular assays can guide selection and design of a limited set of lead biologic candidates, the *in vivo* behavior of drug candidates must subsequently be evaluated in reliable animal models. While non-human primates closely mimic human biology, their use for early development is limited by cost, accessibility, and ethical concerns. Hence, use of smaller animals, like mice, are preferred. However, such studies should be carefully planned and account for cross-species binding and expression differences between mice and humans, such as target recognition of antibody-based biologics. For example, while being a potent vascular endothelial growth factor (VEGF)-blocker in humans, the widely used anti-VEGF human IgG1 bevacizumab is unable to block mouse VEGF, implying that mice could not have been used in its development.^197,198^ Additionally, our understanding of FcRn biology has revealed major differences that must be taken into consideration when conventional mice are used. This is due to large differences in ligand binding to mouse and human FcRn, where mouse IgG binds very weakly to the human form, and human IgG binds stronger to mouse FcRn than to the human counterpart.^199,200^ Despite reports of a correlation between the pharmacokinetics of wild-type human IgG1 variants in conventional mice and humans,^199^ IgG1 variants that have been Fc engineered for enhanced human FcRn engagement and half-life extension fail to engage mouse FcRn efficiently due to loss of pH-dependent binding, resulting in short plasma half-life.^165^ These cross-species differences have motivated the development of mouse strains transgenic for human FcRn and lacking mouse FcRn. These transgenic mice are today the gold standard for pharmacokinetic evaluation of human IgG-based biologics,^201–204^ and importantly, data generated in them correlates with data from both non-human primates and clinical observations.^205,206^

An important, largely overlooked aspect of pharmacokinetic studies of antibodies in mice is their unusually low levels of endogenous IgG, which arises from the pathogen-free housing needed for experimental animal facilities.^201,207,208^ Naturally, injected IgG and albumin-based therapeutics will compete for binding to FcRn in the presence of large amounts of IgG and albumin, which have concentrations of approximately 12 and 40 mg/mL, respectively.^164,209^ This places competitive pressure on the ligand binding sites of FcRn, which modulates the plasma half-life of injected IgG and albumin.^196,201,210,211^ The absence of this pressure may mask relevant differences between candidate biologics,^196^ which should thus be accounted for in order to accurately predict pharmacokinetic properties. This can be achieved by pre-injection of high concentrations of intravenous IgG (IVIg).^201,208^ In addition, advances have recently been made in offering a relevant competitive setting in mice by creating human FcRn transgenic mice that also express human IgG1-Fc.^201,202^ Similarly, there are large differences regarding albumin binding across species. For instance, mouse FcRn binds poorly to human albumin,^200,212^ which effectively prevents human albumin-based formats from being rescued from intracellular degradation. Under these conditions, the plasma half-life of albumin drops to levels similar to irrelevant proteins of a size above the renal clearance threshold.^213^ Using human FcRn transgenic mice may correct for this issue. Additionally, such mice constitutively produce large amounts of endogenous albumin, effectively introducing a competitive environment for human albumin.^196^ In fact, human FcRn binds more efficiently to mouse albumin than the human counterpart,^200,212^ which increases the competitive pressure thathuman albumin-based biologics face in these mice. Alternatives offering a more biologically relevant setting include using human FcRn-expressing mice lacking endogenous albumin, and preloading them with human albumin, much like introducing IVIg when studying IgG,^167,196,204^ or using transgenic mice where mouse albumin has been replaced with the human counterpart.^214,215^

## Cell line development and manufacturing of biologics

Most biologics are expensive therapeutic agents administered directly into the body of patients. Therefore, it is of the utmost importance that they are produced in an efficient, safe, and reproducible manner. The use of mammalian cells for production of marketed antibody therapeutics is most common (www.antibodysociety.org/antibody-therapeutics-product-data), but biologics may also be made in bacteria, yeast, and cell free expression systems.^216^ Production via mammalian cell cultivation most often involves billions of living cells, and it is challenging to control and reliably reproduce the complex biological processes involved at large scales. Therefore, substantial efforts go into the development of ideal cell lines for manufacturing biologics that stably express the product for more than 60 generations at high yields with consistent product quality in a highly reproducible process. As a result of these efforts, the development of production cell lines has been improved over the past decades,^217^ going from random integration of protein-expressing gene(s) followed by extensive screening for high-producing clones^218^ to more targeted approaches, including targeted gene integration for reproducible growth and yield^219,220^ and insertion of larger genetic elements^221^ to obtain robust high producing cell lines, and incorporating numerous cell engineering strategies to gain consistent product quality.^222–226^

Even though high-yielding and robust cell line development approaches have substantially advanced over the past decades, efficient production of biologics remains a continuous challenge. One contributing issue is the increasing complexity of biologics, such as heterologous proteins that can cause added cellular stresses and potentially cell death. Another challenge is the change in expression systems for biologics that often occurs between the early research stage and the later cell line development stage. Transient human expression systems are often preferred early on due to the ease and speed of production of workable product quantities,^227^ while stable CHO cell lines are preferred for commercial production.^9^ This change can cause profound differences in the product yield and quality (e.g., glycosylation, aggregation, protein folding), which means the product needs to be thoroughly re-characterized in the new expression system prior to commercial manufacture. Issues such as low product yield and changes in product quality that may compromise efficacy, quality, and patient safety are frequently encountered during cell line development.

The earlier the cell line development is considered during drug development, the higher the chances are for successful manufacturing. Numerous studies have shown that poor biophysical properties, such as aggregation propensity, are often linked to inefficient production in stable cell lines, underlining the importance of using prediction tools for biophysical properties as an early manufacturability indicator.^93,228^ The chances of successful manufacturing can also be increased by using a robust and flexible cell line development platform that combines early product assessment and stable cell line generation. A targeted integration platform with predictable high yield has strong potential in this regard, where the biologic can be produced in the same cell line from discovery to commercial manufacturing.^219,229–231^ Flexibility can be added by for example having a library of different glycoengineered cell lines,^226^ from where the desired glycosylation profile that determines the biologic’s stability, plasma half-life, and immunogenicity can be investigated and chosen. The cell line that provides the desired glycosylation profile of the product could then be used directly for large-scale manufacturing.

## Antibodies for oral application

To date, most approved biologics are delivered as injectables, and the molecules, therefore, enter the circulatory system and exert their activity there or in tissues. However, alternative approaches are being explored, particularly for combating gastrointestinal (GI) infections.^232–234^ For such biologics, developability aspects are equally important as for injectable molecules. However, while properties such as plasma half-life and immunogenicity are critical factors for intravenously administered biologics, other aspects, such as stability in the GI tract and shelf-life are more important for orally available biologics that exert their function in the GI tract. Moreover, different quality parameters, such as product purity and presence of other proteins, may potentially be of less concern for orally administered biologics, as the GI tract normally encounters a wide range of macromolecules. As an example of how biologics can be optimized for the oral route, Fiil et al.^235^ engineered a highly biophysically stable homodivalent V_H_H construct for feed applications and demonstrated its functionality in inhibiting proliferation of enterotoxigenic *E. coli* (ETEC) in piglets.^235^ A key factor in their early design was the choice of a linker that was surprisingly more stable under GI conditions compared to the more natural hinge region of IgG3, which had previously been used as a linker for other orally delivered proteins.^235,236^ Similarly, Virdi et al. experimented with other formats, such as IgA-like molecules, which were also demonstrated to be effective in inhibiting ETEC proliferation in the GI tract of piglets.^237^ In this case, it was speculated that the incorporation of an Fc region would increase the retention time in the GI tract, which is conceptually similar to extending half-life.

In some cases, the biophysical stability of a biologic can be further optimized during the discovery process or via subsequent protein engineering efforts. In the case of nanobodies, one approach involves intracellular selection, which has been shown to select for nanobodies with higher stability.^238^ This can be followed with experiments in which nanobodies are subjected to elevated temperatures before the screening process, enabling the identification of nanobodies with higher refolding capacity,^239^ as well as protein engineering efforts where nanobodies are further stabilized against high temperature and proteases via the introduction of additional disulfide bonds between opposing beta strands.^240–242^

Finally, when a biologic is to be delivered orally, the use of alternative expression systems, such as microbial fermentation, the use of algae or transgenic plants, or even *in situ* production by engineered cells, may be considered.^243–246^ Such systems can potentially reduce both cost and time for production of the biologics, which could expand the range of applications for such molecules.

## Concluding remarks

Recently, a shift has occurred within the discovery and development of biologics, from mainly focusing on high affinity and specificity to the target, hitting the right epitope, and conveying the desired function, to now also taking developability aspects into consideration. This broadened discovery and multidimensional engineering mindset is likely to yield better drug candidates, as well as reducing the number of late-stage failures during drug development. However, in many cases, especially with completely novel types of biologics, it is not always clear what constitutes a good developability profile, although some examples of such profiles (*e.g*., marketed drug-likeness^79^ and the Therapeutic Antibody Profiler^80^) are beginning to emerge. With more sequence information and biophysical data becoming publicly available, the task of establishing guiding principles on developability is becoming more approachable. Within this field, we expect that *in silico* predictions will play an increasingly larger role early in the discovery process, as they allow for very high-throughput analysis at low cost (when established). However, using *in silico* models may come with some inherent uncertainties due to biases in existing datasets, and re-evaluating algorithms and both expanding existing and building new datasets will continue to be important. During generation of these datasets, it is likely beneficial to include and explore molecules that may not be predicted to have superior developability profiles.

Another complication is the fact that some biophysical properties are inherently dynamic, such as aggregation and partial unfolding, and it can be important that *in silico* methods are able to incorporate molecular behavior in their prediction. As an example, as structure models are not always exact, simulations that take this into account are likely needed as a complement to steady-state models to improve the accuracy and reliability of biophysical predictions. To further improve the *in silico* approaches for the development of optimal biologics, both knowledge-based and physics-based methods are needed. Another hurdle for development of reliable *in silico* models is the lack of self-consistent and reproducible data obtained from experiments performed on a large number of molecules performed under similar conditions using similar protocols and instruments. The ongoing digital transformation of the biopharmaceutical industry is expected to ameliorate this difficulty and facilitate the generation of improved artificial intelligence and machine learning methods. Finally, the formation of collaborative consortia between industry and academia in a pre-competitive space to make self-consistent and reproducible data available for machine learning will be another great step forward in the improvement of *in silico* approaches and models.

In addition to *in silico* methods, we foresee that biophysical methods will continue to play a role to assess developability measures. Automation, miniaturization, and digitalization are key trends within drug development. Combined with novel *in vitro* assays, this may enable much more powerful and intelligent screening and characterization early in the discovery process for new biologics. However, a challenge in this area remains. While having a powerful discovery engine can be a major advantage for developing new biologics, it is both complicated and expensive to build up such capacity and educate personnel in the use of advanced systems. Moreover, as medicines are becoming increasingly more tailored and personalized, versatility and modularity of large discovery platforms need to be improved, so that they are not only optimized to discover modalities against a single type of indication.

Another avenue that will aid the development of biologics is the generation and use of animal models that better reflect the clinical setting and how the biologic performs in humans. In this regard, some important aspects include pharmacokinetics, immunogenicity, efficacy, and engagement of effector functions.

As biologics are often complex molecules to manufacture, it is of high importance that expression systems and purification methods are optimized to enable repeated, reproducible production of high-quality material. This involves optimization of yields, folding, post-translational modifications, such as glycosylation, and reduction of host cell proteins. Here, it is expected that glycoengineering will continue to play an important role, and we foresee that the consideration of glycosylation patterns (and other post-translational modifications) early in the discovery process might improve success rates for many protein-based biologics. In this area, however, much remains unknown, and it will be important to better establish knowledge and guidelines for how not only to engineer biologics to have human-like post-translational modifications, but also to have modifications that are even better than the corresponding human ones.

As a final remark, it is worth mentioning that most biologics to date are administered as injectables. In the future, it is expected that more biologics will be delivered orally, by the pulmonary route, or by other routes. This will undoubtedly have an influence on how new biologics should be developed and formulated, and may allow use of entirely new production systems.

## Abbreviations

AC-SINS: Affinity-capture self-interaction nanoparticle spectroscopy
ADA: Anti-drug antibodies
APC: Antigen presenting cell
CDR: Complementary-determining region
CHO: Chinese hamster ovary
CIC: Cross-interaction chromatography
CMC: Chemistry, manufacturing, and control
CS-SINS: Charge-stabilized self-interaction nanoparticle spectroscopy
ETEC: Enterotoxigenic E. coli
FcRn: Neonatal Fc receptor
Fvs: Fragment variable regions
GI: Gastrointestinal
HERA: Human endothelial cell-based recycling assay
HIC: Hydrophobic interaction chromatography
KLH: Keyhole limpet hemocyanin
LPS: Lipopolysaccharide
MD: Molecular dynamics
PBMC: Peripheral blood mononuclear cell
PSP: Polyspecificity particle assay
PSR: Polyspecificity Reagent
SIC: Self-interaction chromatography
SMAC: Standup monolayer chromatography
VH: Variable heavy
VL: Variable light
